# Supplementary material for: Preparation of Nanoparticle-Loaded Extracellular Vesicles Using Direct Flow Filtration
Source: Pharmaceutics. 2023 May 20;15(5):1551. doi: 10.3390/pharmaceutics15051551 (PMC10221999; doi:10.3390/pharmaceutics15051551)
Supplement: Supplementary file 1 [file pharmaceutics-15-01551-s001.zip › pharmaceutics-2390658-supplementary.pdf]

Electronic Supporting information

PPreparation of Nanoparticle-Loaded Extracellular Vesicles Using Direct Flow Filtration

Shomit Mansur, Shahriar Habib, Mikayla Hawkins, Spenser R. Brown, Steven Weinman *, and Yuping Bao *

Chemical & Biological Engineering, The University of Alabama, Tuscaloosa, AL 35487, USA
* Correspondence: stweinman@eng.ua.edu (S.W.); ybao@eng.ua.edu (Y.B.)

1. FTIR of quercetin

The Quercetin and iron complexation is mainly associated with the hydroxyl groups or the ketocarbonyl group. Therefore, the analysis was focused on the range of 400-2000 cm^{-1}. Figure S1 shows the full ATR-FTIR scans of quercetin and quercetin iron complex nanoparticles (QFeNPs).

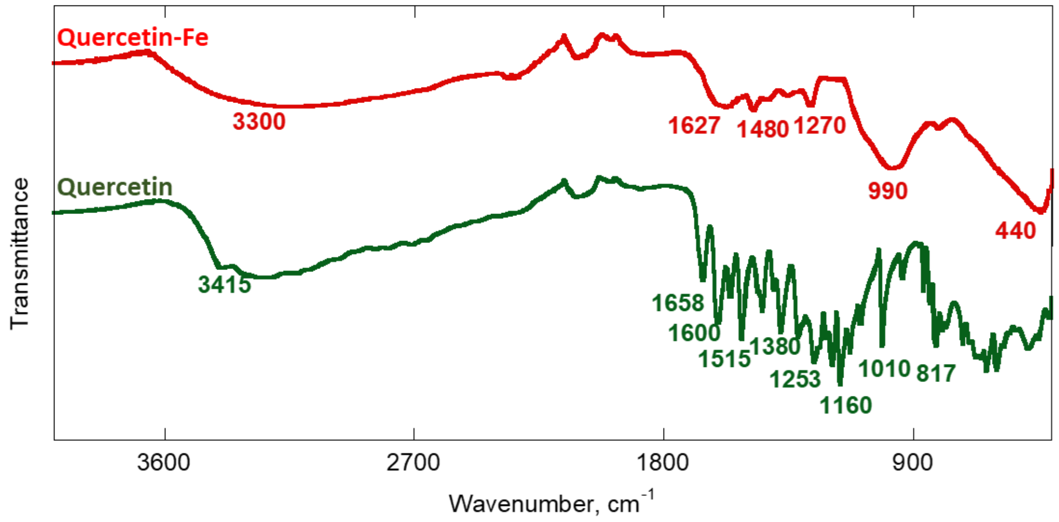

Figure S1 : ATR-FTIR spectra of pure quercetin and quercetin iron complex nanoparticles. Range 400-4000 cm^{-1}

2. Protein quantification from two cycles of centrifugation

To verify whether the second centrifugation removed the majority of cellular proteins, we have performed a Bradford assay to quantify the total proteins before and after the centrifugation cycle (10,000 g, 10 min). Briefly, after collecting 3 mL of serum-free media solution in a 50 mL centrifuge tube, it was diluted up to 20 mL using 1X PBS following our standard protocol for EV isolation using DFF. In order to separate cell debris and cellular proteins, we performed two stages of centrifugation (1000 g for 10 min and 10,000 g for

10 min) at 4 ºC. 500 μL of supernatant from each centrifugation cycle was separated for Bradford assay to determine whether proteins were separated out during the centrifugation cycles. The supernatant solutions were diluted 5x by adding 400 μL DI water to 100 μL of the solution. Then the solution was mixed with 200 μL of Bradford reagent. Absorbance at 595 nm was measured after 10 minutes using Eppendorf Biospectrometer model no. 6136000851, and the protein concentration was determined after obtaining a standard curve for BSA. The difference in protein concentrations before and after centrifugation suggested the removal of majority protein particles (Table S1).

Table S1. Protein concentration before and after centrifugation

Sample	A595	ug/ml Protein
**Pre-Centrifugation**	0.47	11.00612
**Post-Centrifugation**	0.348	7.948762
	**Difference** =	3.05736

3. EVs isolation via ultracentrifugation

To compare the effectiveness of ultrafiltration with direct flow filtration in EV isolation, EVs also were prepared using a series of centrifugation processes. Specifically, 3 mL of serum-free growth media was first collected after 24 h incubation and then centrifuged at 1,000 g for 5 min at 4 °C to remove cell debris. The supernatant was collected and centrifuged again at 10,000 g for 10 min at 4 °C to remove large cellular proteins. Then, the supernatant was filtered using a 0.2 μm cellulose acetate membrane syringe filter to separate EVs and other molecules of sizes above 200 nm and then it was centrifuged at max speed (50,000 g) for 30 min at 4 °C. The pellet after centrifugation was collected and redispersed in 2 mL PBS and was extruded through 200 nm membrane filter 5 times using Avanti extruder.

Figure S2 shows the typical TEM images at different magnifications and DLS plot of NP-loaded EVs isolated by ultracentrifugation. Compared to the NP-loaded EVs isolated by direct flow filtration (Figure 2A), the EVs isolated by ultracentrifugation were larger in size (> 500 nm) with a larger size variation (Figure S2A). Figure S2B shows the TEM image of EVs from Figure S2A at a higher magnification. The DLS plot showed a broader size distribution with an average size distribution around 344 nm, but a shoulder peak around 100 nm could be seen clearly and the tail to larger size almost to 900 nm (Figure S2C).

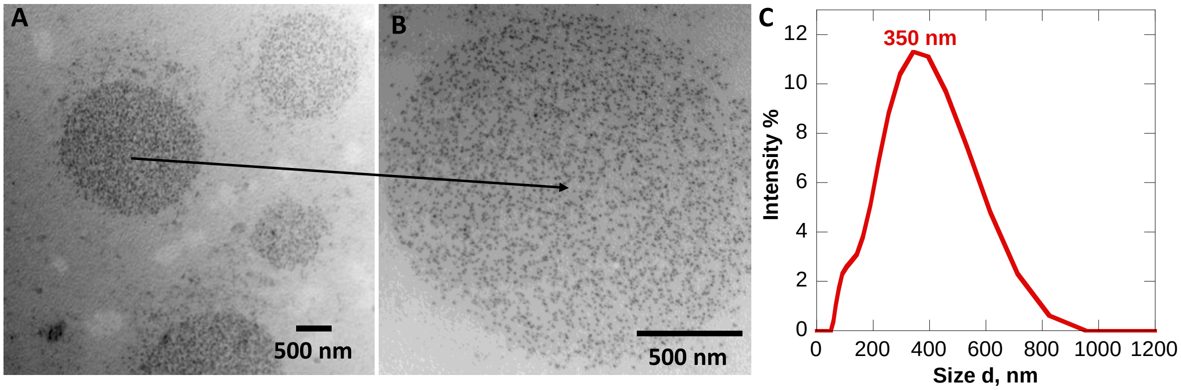

Figure S2: NP-loaded EVs isolated by ultracentrifugation method: (A & B) Representative TEM images at different magnifications and (C) DLS plot.

Ultracentrifugation was also used to isolate empty EVs from cells. Figure S3 shows the typical TEM image of empty EVs isolated by ultracentrifugation method, where the EVs were stained with 2% uranyl acetate. Compared to the empty EVs isolated by direct flow filtration (Figure 3A), the EVs isolated by ultracentrifugation were larger in size with a larger size variation (Figure S3). Unfortunately, a quality DLS plot could not be obtained due to the low EV concentration and polydispersity

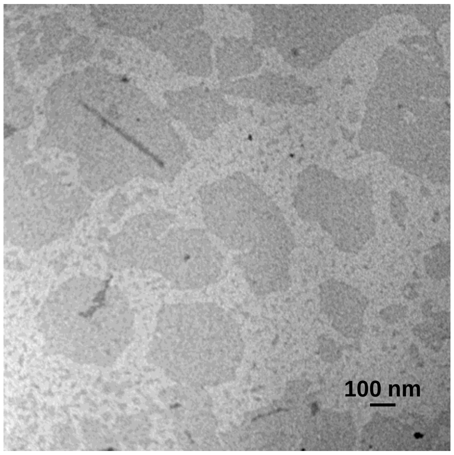

Figure S3: TEM image of empty EVs isolated by ultracentrifugation method.

4. EVs formation at different conditions

To rule out apoptotic body formation at the stressed condition, we have taken images of the cells treated with NPs after 24 hours under healthy condition (regular growth media) and serum-free condition (Figure S3 A &B). Specifically, MDA-MB-231Br cells were seeded in two T-25 flasks with 3 mL of DMEM media with supplements 10% FBS and 1% penicillin/streptomycin and constantly checked for confluency every

24 hour. Cell count was $3x10^5$ each. When cells reached 50% confluency, cells were treated with NPs at a final concentration of 200 μg/mL. After 24 h incubation, the growth media were removed for both flasks, but one flask was added with 3 mL of serum-free growth media containing no supplements while the other flask was added 3 mL of new growth media with same supplements. After an additional 24 h, the cells from both flasks were imaged using Ts2R Nikon inverse compound light microscope. With growth media (with/without serum) remaining in the cell growing flasks, not much difference in cell morphologies were observed for cells under those two conditions. However, after removing the growth media, the cells under the stressed condition seemed shrank, but remained attached to the flask (Figure S4 C&D). This observation suggested that the cells did not go through apoptosis and generate apoptotic bodies.

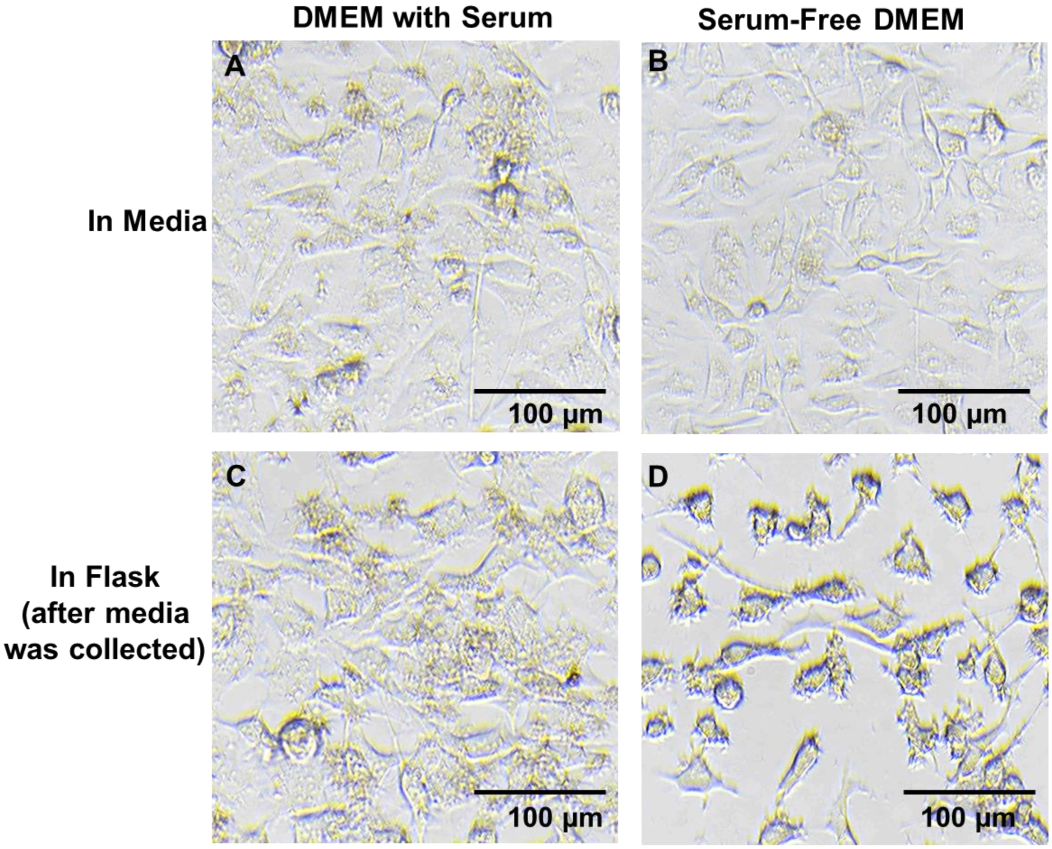

Figure S4: Bright field images of cells treated with NPs under healthy condition: (A) in growth medium, and (C) after medium removal, and stressed serum-free condition: (B) in growth medium, and (D) after medium removal.
